# Microbiologically influenced corrosion of FeCoNiCrMn high-entropy alloys by *Pseudomonas aeruginosa* biofilm

**DOI:** 10.3389/fmicb.2022.1009310

**Published:** 2022-10-10

**Authors:** Jike Yang, Yu Zhang, Weiwei Chang, Yuntian Lou, Hongchang Qian

**Affiliations:** ^1^Beijing Advanced Innovation Center for Materials Genome Engineering, Institute for Advanced Materials and Technology, University of Science and Technology Beijing, Beijing, China; ^2^National Materials Corrosion and Protection Data Center, University of Science and Technology Beijing, Beijing, China; ^3^BRI Southeast Asia Network for Corrosion and Protection (MOE), Shunde Innovation School, University of Science and Technology Beijing, Foshan, China

**Keywords:** high-entropy alloys, microbiologically influenced corrosion, *Pseudomonas aeruginosa*, biofilm, passive film

## Abstract

*Pseudomonas aeruginosa* is widely found in industrial water and seawater. Microbiologically influenced corrosion (MIC) caused by *P. aeruginosa* is a serious threat and damage to the safe service of steel materials. In this study, the MIC behavior of FeCoNiCrMn high-entropy alloy (HEA) by *P. aeruginosa* biofilm was investigated in the simulated marine medium. The maximum pitting depth of the HEA coupons in the *P. aeruginosa*-inoculated medium was ~4.77 μm, which was 1.5 times that in the sterile medium. EIS and potentiodynamic polarization results indicated that *P. aeruginosa* biofilm reduced the corrosion resistance of the passive film of HEA coupons and promoted its anodic dissolution process. XPS and AES results further demonstrated that *P. aeruginosa* interfered with the distribution of elements in the passive film and significantly promoted the dissolution of Fe.

## Introduction

Microbiologically influenced corrosion (MIC) refers to the phenomenon that the metabolic activities of microorganisms and their products interact with metals in the process of corrosion reaction to affect the cathodic or anodic process, thereby affecting the behavior and mechanism of metal corrosion (Liu and Cheng, 2018; Cai et al., 2021; Li et al., 2021; Qian et al., 2021a,b; Cui et al., 2022). At present, most of researches on MIC is analyzed and explained from a macroscopic level (Little et al., 2020; Qian et al., 2022a,b). By exploring the adhesion behavior of microorganisms/biofilms on the surface of the sample and the changes in the metal surface composition and microenvironment after corrosion, corresponding to the surface morphology and electrochemical data, the corrosion mechanism can be analyzed and speculated.

*Pseudomonas aeruginosa*, a long rod-shaped Gram-negative bacterium, widely exists in soil, swamp, and marine environment (Murie et al., 2019). Because of the ability to release organic acids, carbon dioxide and sulfate during the process of metabolism, it is regarded as a typical corrosive microorganism and can act as a pioneer to adhere to different types of material surfaces among many environmental microorganisms (Liu et al., 2021). Jia et al. found that *P. aeruginosa* could accelerate the pitting corrosion of 304 stainless steel under the anaerobic condition, with a maximum pitting depth of 7.4 μm after 14 days of immersion (Jia et al., 2017). Hamzah et al. demonstrated that *P. aeruginosa* in seawater significantly accelerated the corrosion rate of carbon steel, 1.6 times that of the sterile group, and the composition of corrosion products also changed greatly due to the metabolism of *P. aeruginosa* (Hamzah et al., 2014). Recent studies have shown that *P. aeruginosa* can also utilize extracellular electron transfer (EET) to promote MIC (Li Z. et al., 2022). Huang et al. demonstrated that *P. aeruginosa*-secreted electron mediator PYO promoted EET to accelerate the degradation of 304 stainless steel passive film, in which iron oxide dissolution was significantly accelerated (Huang et al., 2022).

High-entropy alloys (HEAs), a new class of materials that differ from traditional ones consisting of one or two main components, are alloys formed by mixing equal or relatively large proportions of five or more elements (Qiu et al., 2017; Senkov et al., 2018; George et al., 2019). Among many HEAs systems, the alloy composed of five components of Fe, Co, Ni, Cr, and Mn can form a single-phase face-centered cubic (FCC) structure at room temperature, also known as Cantor alloy (Cantor, 2021; Qian et al., 2021a,b). The corrosion behavior of HEAs have been studied in a variety of environments over the past decade (Chen et al., 2005; Liu et al., 2015; Qiu et al., 2015; Fu et al., 2021). Kai et al. studied the corrosion behavior of Cantor alloy at 700°C and 950°C in different ratios of CO_2_/CO mixtures. The results demonstrated that the thinner oxide layer was mainly composed of MnO, (Mn, Fe)_3_O_4_, and (Mn, Cr)_3_O_4_ at 700°C. While at 900°C a double oxide layer was formed with (Mn, Fe)_3_O_4_ and MnO as the outer layer, (Mn, Cr)_3_O_4_ and MnO as the inner layer (Kai et al., 2019). Cui et al. investigated that anti-corrosion property of Cantor alloy coatings prepared by high-speed laser cladding (HSLC) and ultrasonic surface mechanical rolling treatment (SMRT). The results suggested that the Cantor alloy coatings after SMRT presented a gradient structure, which was planar crystal, columnar dendrites and equiaxed crystals from inside to outside, and the corrosion resistance was better than that without SMRT in 3.5% NaCl solution (Cui et al., 2020). However, few studies on MIC of Cantor alloy have been reported. Herein, we systematically investigated the MIC behavior of Cantor alloy under the intervention of *P. aeruginosa* in the simulated marine medium.

## Materials and methods

### Bacteria, medium, and coupon

Marine *P. aeruginosa* (MCCC 1A00099) was obtained from the Marine Culture Collection of China (MCCC). The composition of the 2216E medium used for bacterial cultivation and all examinations was as follows (g/L): 5.0 peptone, 1.0 yeast extract, 0.1 ferric citrate, 19.45 NaCl, 5.98 MgCl_2_, 3.24 Na_2_SO_4_, 1.8 CaCl_2_, 0.55 KCl, 0.16 Na_2_CO_3_, 0.08 KBr, 0.034 SrCl_2_, 0.08 SrBr_2_, 0.022 H_3_BO_3_, 0.004 NaSiO_3_, 0.0024 NaF, 0.0016 NH_4_NO_3_, and 0.008 NaH_2_PO_4_. The 2216E medium was autoclaved at 121°C for 20 min before use (MLS-3781l-PC, Panasonic). The initial concentration of *P. aeruginosa* was set to 10^6^ cells/ml by a hemocytometer under an optical microscope (Lab A1, Zeiss) at 400× magnification. The equiatomic FeCoNiCrMn HEA was cast using arc melting under high-purity argon protection. The HEAs were machined to a size of 10 mm × 10 mm × 3 mm coupons for all MIC measurements. The coupons were abraded with sandpapers in order of 400#, 600#, and 800#, and then ultrasonically cleaned with absolute ethanol. After nitrogen drying, all coupons were exposed to ultraviolet light for more than 30 min.

### Surface characterization

Scanning electron microscopy (SEM, SU8010, Hitachi) was used to observe the morphology of *P. aeruginosa* attached to the coupon surface. After immersion, the coupons were transferred to 2.5% glutaraldehyde solution overnight at 4°C in order to fix *P. aeruginosa* biofilm. Next, dehydration with a gradient of ethanol solutions (50, 70, 90, 95, 100 vol.%) was performed (Li et al., 2020). Before SEM observation, the coupons were sputtered with Au to improve surface conductivity. The live and dead state of cells in biofilm was quantified using confocal laser scanning microscopy (CLSM, TCS SP8, Leica). Fluorescent stains SYTO-9 and propidium iodide (PI) dyes (LIVE/DEAD™ BacLight™ Bacterial Viability Kit, Thermo Fisher) were used to distinguish between live and dead bacteria, showing green and red, respectively. The characterization of corrosion pits on the coupon surface was measured by CLSM (VK-X250K, KEYENCE). The composition and thickness of the HEA passive film were characterized *via* X-ray photoelectron spectroscopy (XPS, ESCALAB 250Xi, Thermo Fisher) and Auger electron spectroscopy (AES, PHI-700, ULVAC-PHI). The dissolved metallic ions were evaluated using inductively coupled plasma mass spectrometry (ICP-MS, iCAP TQs ICP-MS, Thermo Fisher), and the detailed process was described as reported previously (Chang et al., 2022).

### Electrochemical measurements

The traditional three-electrode system combined with an electrochemical workstation (Reference 600 plus, Gamry) was used to perform the electrochemical measurements, including open circuit potential (OCP), electrochemical impedance spectroscopy (EIS), linear polarization resistance (LPR), and potential polarization. The HEA coupons (1 cm^2^) were used as the working electrode, platinum foil as the counter electrode, and saturated calomel electrode (SCE) as the reference electrode. Before all electrochemical texts, the electrochemical cells were autoclaved at 121°C for 20 min, and the electrodes were sterilized by isopropanol and ultraviolet irradiation for 30 min. LPR test was conducted from −10 to 10 mV vs. *E*_OCP_ at a scanning rate of 0.125 mV/s. EIS test was performed at the *E*_OCP_ with a sinusoidal AC voltage of 5 mV at 10^−2^ to 10^5^ Hz, and the results were analyzed with ZSimDemo. Potentiodynamic polarization curves were obtained after 14 days of immersion at 0.1667 mV/s, starting from −250 mV vs. *E*_OCP_ to the final potential corresponding to a current density of 1 mA/cm^2^ (Dai et al., 2020a,b).

## Results and discussion

### Biofilm characterization

**Figure 1:**
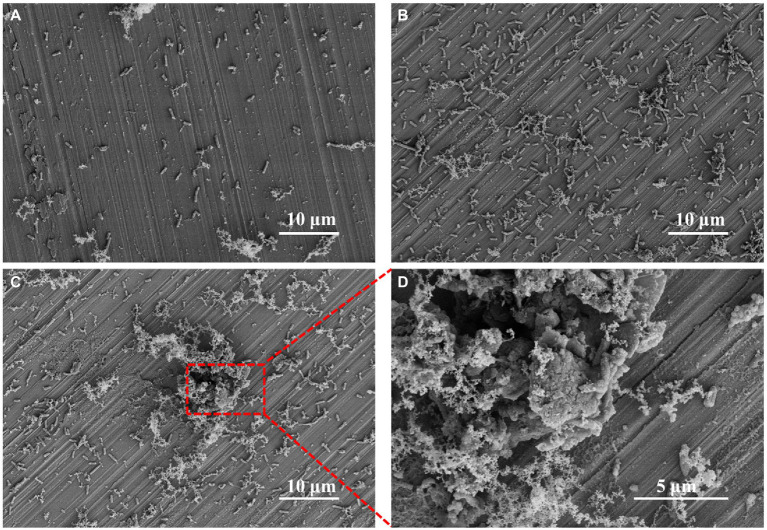
SEM images of *Pseudomonas aeruginosa* on HEA coupon surfaces after **(A)** 3 days, **(B)** 7 days, **(C)** 14 days and **(D)** the enlarged image of the figure **C**.

The sterile FeCoNiCrMn HEA was immersed in 2216E containing *P. aeruginosa* for 3, 7, and 14 days, respectively. The surface morphology of HEA coupons was shown in Figure 1. After 3 days, rod-shaped *P. aeruginosa* were uniformly distributed on the coupon surface, accompanied by local accumulation of metabolites (Figure 1A). After 7 days, the number of attached *P. aeruginosa* increased significantly and gathered into clusters, which already had the rudiment of biofilm (Figure 1B). With the further immersion, mature biofilm was observed on the coupon surface after 14 days (Figure 1C). Bacteria were wrapped by fibrous EPS, which promoted aggregation among bacteria and constituted a complete three-dimensional structure to resist the external adverse environment (Di Martino, 2018; Karygianni et al., 2020). In Figure 1D, further magnification of the biofilm revealed that bacteria were coated with metabolites and mixed with corrosion products. The above results indicated that the formation of biofilm greatly changed the original state of coupon surface and may cause MIC (Qian et al., 2022a,b).

**Figure 2:**
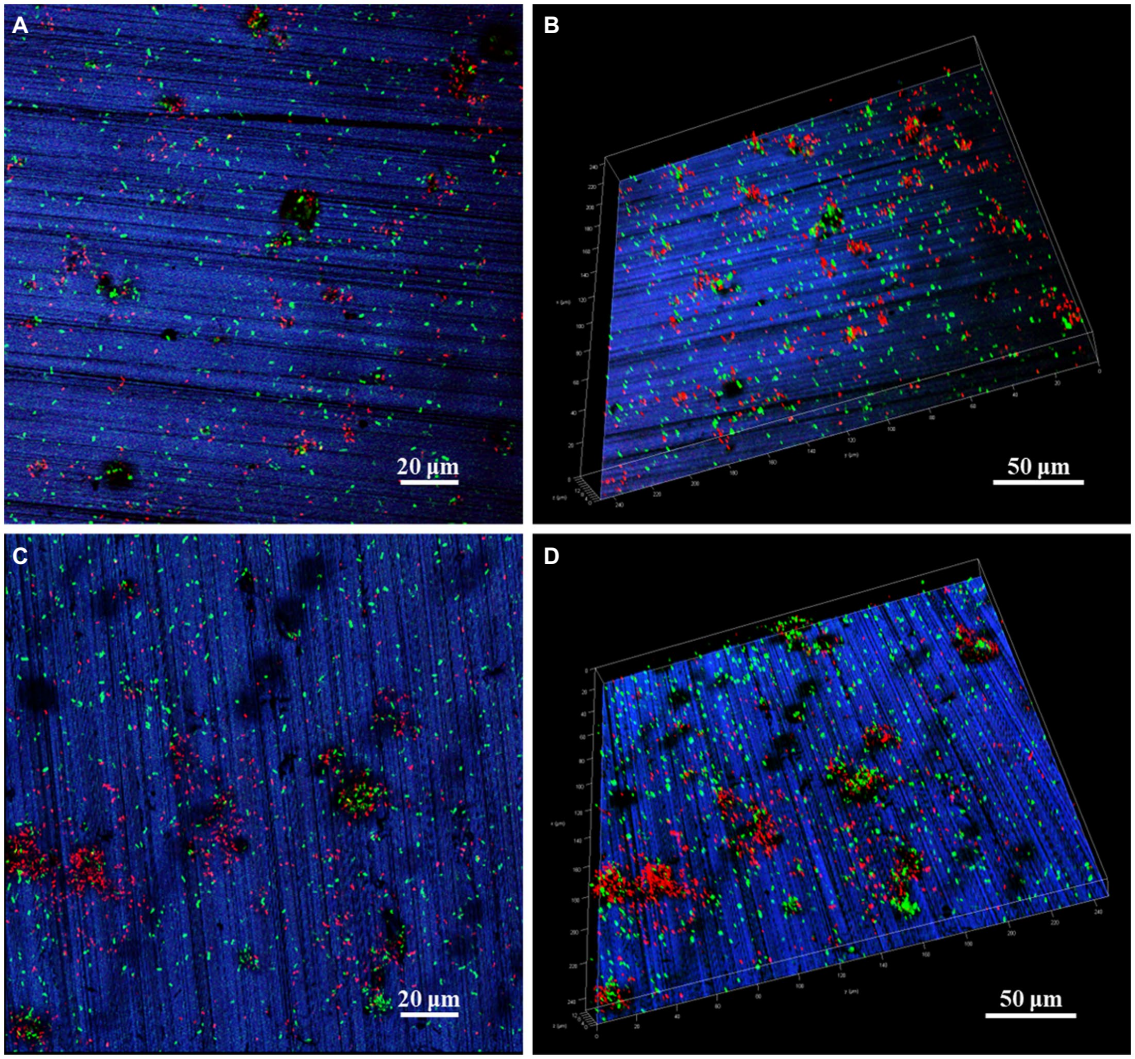
CLSM images of *P. aeruginosa* biofilm on HEA coupon surfaces after **(A,B)** 7 days and **(C,D)** 14 days.

The *P. aeruginosa* distribution and biofilm coverage were further observed by CLSM. After 7 days of immersion, the bacteria were evenly distributed on the coupon surface. Some dead bacterial cells (red) appeared locally, and the scratches produced by pre-machining were clearly visible (Figures 2A,B). After 14 days of immersion, the large bacterial clusters contained more dead bacteria, while the newly formed smaller clusters appeared around them, indicating that the biofilm on HEA coupon surface presented a dynamic alternating state (Figures 2C,D). Compared with the HEA coupons immersed for 7 days, more black spots were observed on the 14th day, and most of them appeared under the biofilm, which may be caused by local corrosion (Liu et al., 2017; Li J. et al., 2022). Combined with the results in Figures 1, 2, it was shown that *P. aeruginosa* could attach to the coupon surface and form biofilm during the 14-day immersing period, and the local corrosion tendency was obvious.

### Pit morphology and depth

**Figure 3:**
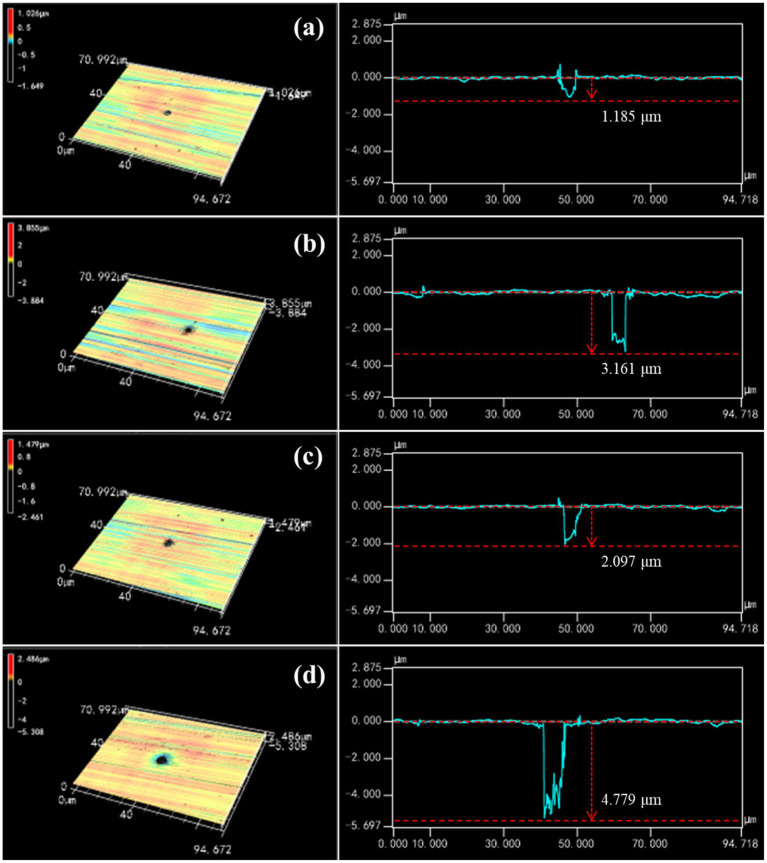
The characteristics of the largest pit found on the HEA coupon surface after immersion: sterile medium for **(A)** 7 days and **(B)** 14 days; *P. aeruginosa*-inoculated medium for **(C)** 7 days and **(D)** 14 days.

To further explore the influence of *P. aeruginosa* biofilm on HEA coupons, the morphology and depth of the largest pit were observed by CLSM after immersion in the sterile and *P. aeruginosa*-inoculated medium for 7 and 14 days respectively, as shown in Figure 3. After 7 days, typical pitting morphology appeared on the HEA coupons in sterile and inoculated medium. The pit depth of the HEA coupon in the inoculated medium was 2.09 μm, almost twice that of the sterile medium (1.18 μm). With the increasing immersion time, the pit depth of the *P. aeruginosa*-inoculated medium reached 4.77 μm on the 14th day, which was much deeper than the 3.16 μm of the sterile medium. The above results indicated that the initiation and development of pitting corrosion on the HEA coupons were promoted by the adhesion of *P. aeruginosa* biofilm.

### Electrochemical measurements

**Figure 4:**
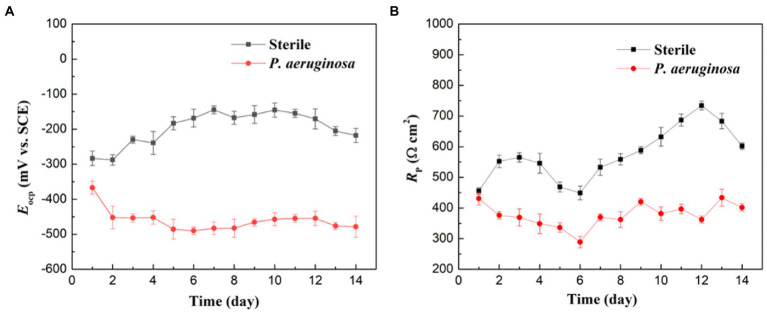
**(A)** OCP and **(B)** LPR measurements of the HEA coupons after immersion in sterile and *P. aeruginosa*-inoculated medium for 14 days.

Electrochemical measurements were performed to investigate the MIC behaviors of the HEA. In Figure 4A shows the variation of *E*_OCP_ of the HEA coupons in the sterile and *P. aeruginosa*-inoculated medium during 14 days of immersion. The variation of *E*_OCP_ in the sterile medium showed a trend of first increasing and then decreasing, and kept above −300 mV as a whole. During the first 7 days of immersion, *E*_OCP_ slightly increased from −283 mV to the maximum −144 mV, then began to decline, and finally stabilized at −217 mV on the 14th day. In contrast, *E*_OCP_ of the HEA coupons in *P. aeruginosa*-inoculated medium dropped rapidly from −367 mV to −452 mV on the 2nd day and then remained roughly stable around −500 mV. These results suggested that the corrosion thermodynamic trend of HEA increased significantly due to the presence of *P. aeruginosa*.

Figure 4B shows the variations of *R*_p_ values of the HEA coupons in the sterile and *P. aeruginosa*-inoculated medium during 14 days of immersion. In the sterile medium, the *R*_p_ values maintained an upward trend during the whole immersion period, and only recovered rapidly after a drop between the 4th and 7th days, which may be caused by the rapid repair of the passive film after metastable pitting corrosion (Hou et al., 2020; Zhu et al., 2022). In the *P. aeruginosa*-inoculated medium, *R*_p_ values were much lower than that in sterile medium during the 14-day immersion. The above results indicated that *P. aeruginosa* accelerated the corrosion of HEA.

**Figure 5:**
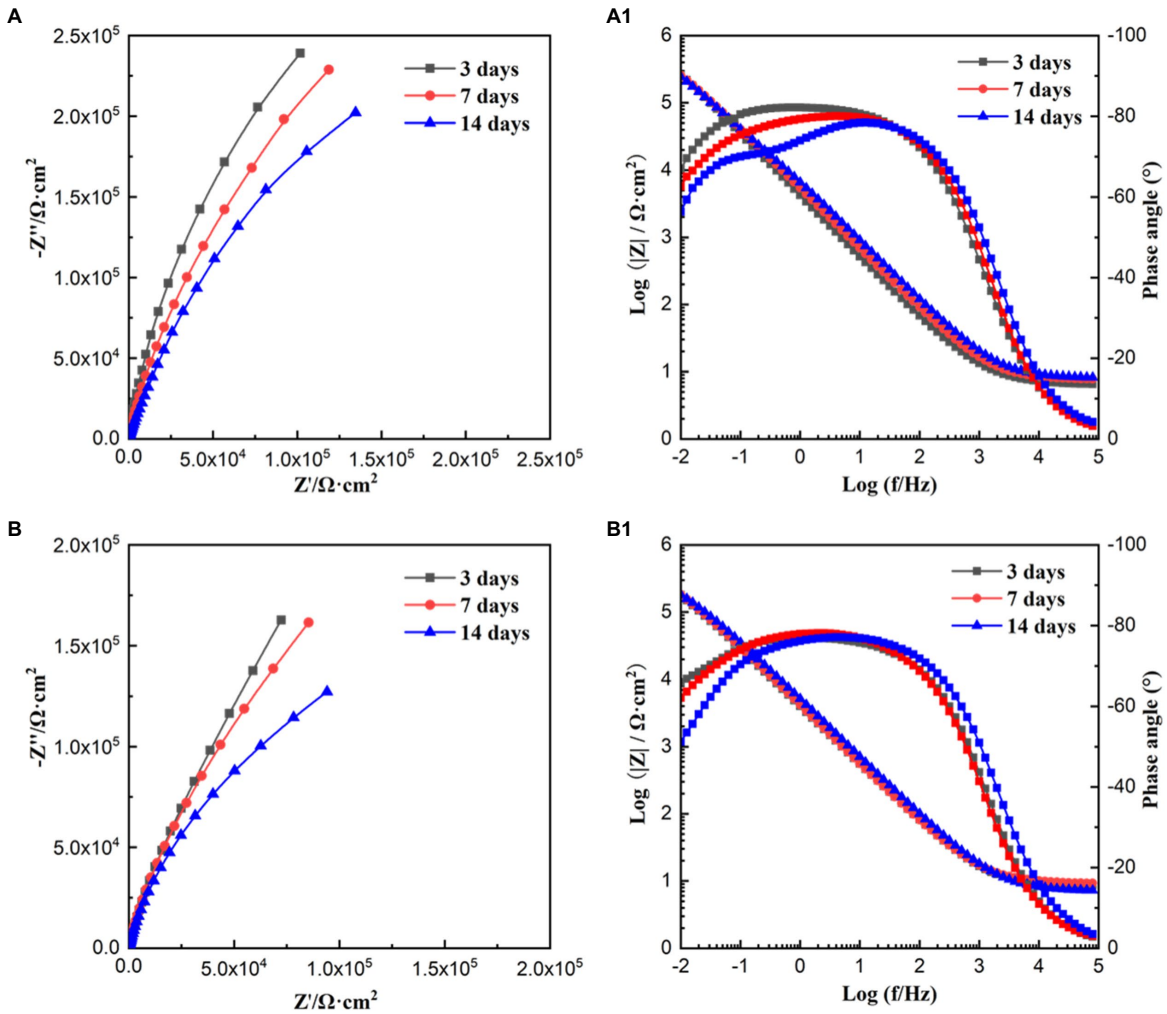
Nyquist and Bode plots obtained for HEA coupons in sterile **(A,A1)** and *P. aeruginosa*-inoculated medium **(B,B1)** for 3, 7, and 14 days, respectively.

Figure 5 shows the EIS results under the sterile and *P. aeruginosa*-inoculated conditions during 14-day immersion. According to Figures 5A,B, the diameter of the impedance loops in the Nyquist plots for the sterile and *P. aeruginosa*-inoculated decreased gradually with the extension of immersion time. In comparison, the diameter in low frequency region decreased more obviously under the effect of *P. aeruginosa* biofilm. Moreover, the diameter of impedance loop in the *P. aeruginosa*-inoculated medium was significantly smaller than that in the sterile medium on the 14th day, indicating that the passive film of HEA coupons was damaged and the corrosion was further accelerated due to the presence of *P. aeruginosa* biofilm. According to Figures 5A1,B1, wider peaks can be observed in the Bode diagram regardless of the presence of *P. aeruginosa* biofilm, probably formed by the merger of peaks in the low and intermediate frequency regions, suggesting that there were two time constants.

**Figure 6:**
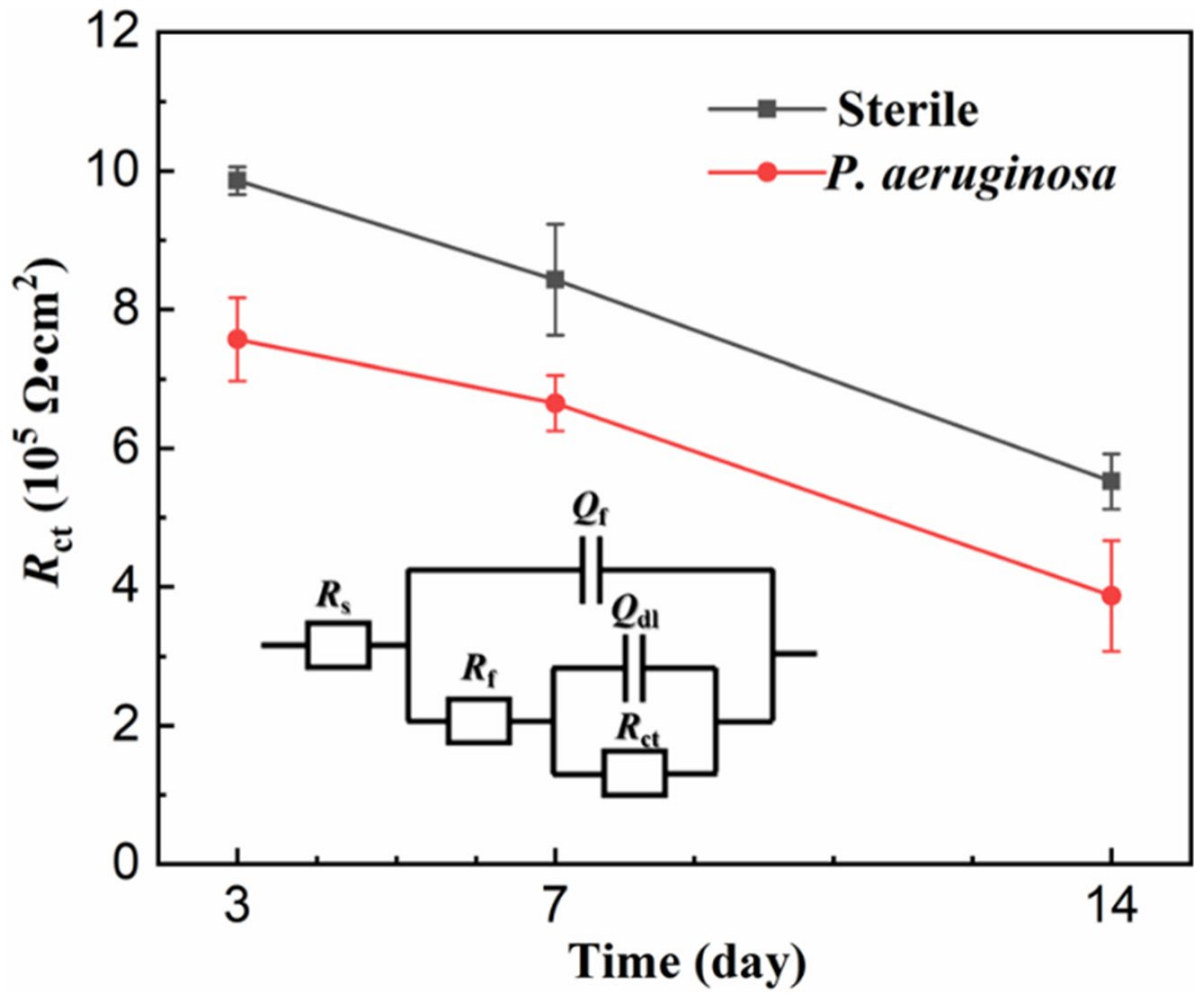
Equivalent electrical circuits simulating the EIS diagrams.

The EIS results were analyzed by the equivalent electrical circuit, as shown in Figure 6. *R*_s_ represent the resistance of the solution, *Q*_f_ and *R*_f_ stand for the capacitance and resistance of the biofilm and corrosion products layer, and *Q*_dl_ and *R*_ct_ are the capacitance and charge transfer resistance of the double layer. *R*_ct_ is inversely proportional to the corrosion rate and is often used as an important indicator to evaluate MIC (Batmanghelich et al., 2017). With the extension of immersion time, the *R*_ct_ values of the sterile and *P. aeruginosa*-inoculated conditions gradually decreased. A more significant decrease was observed in the *P. aeruginosa*-inoculated medium, indicating that the corrosion of HEA coupons was accelerated due to the *P. aeruginosa* biofilm.

**Figure 7:**
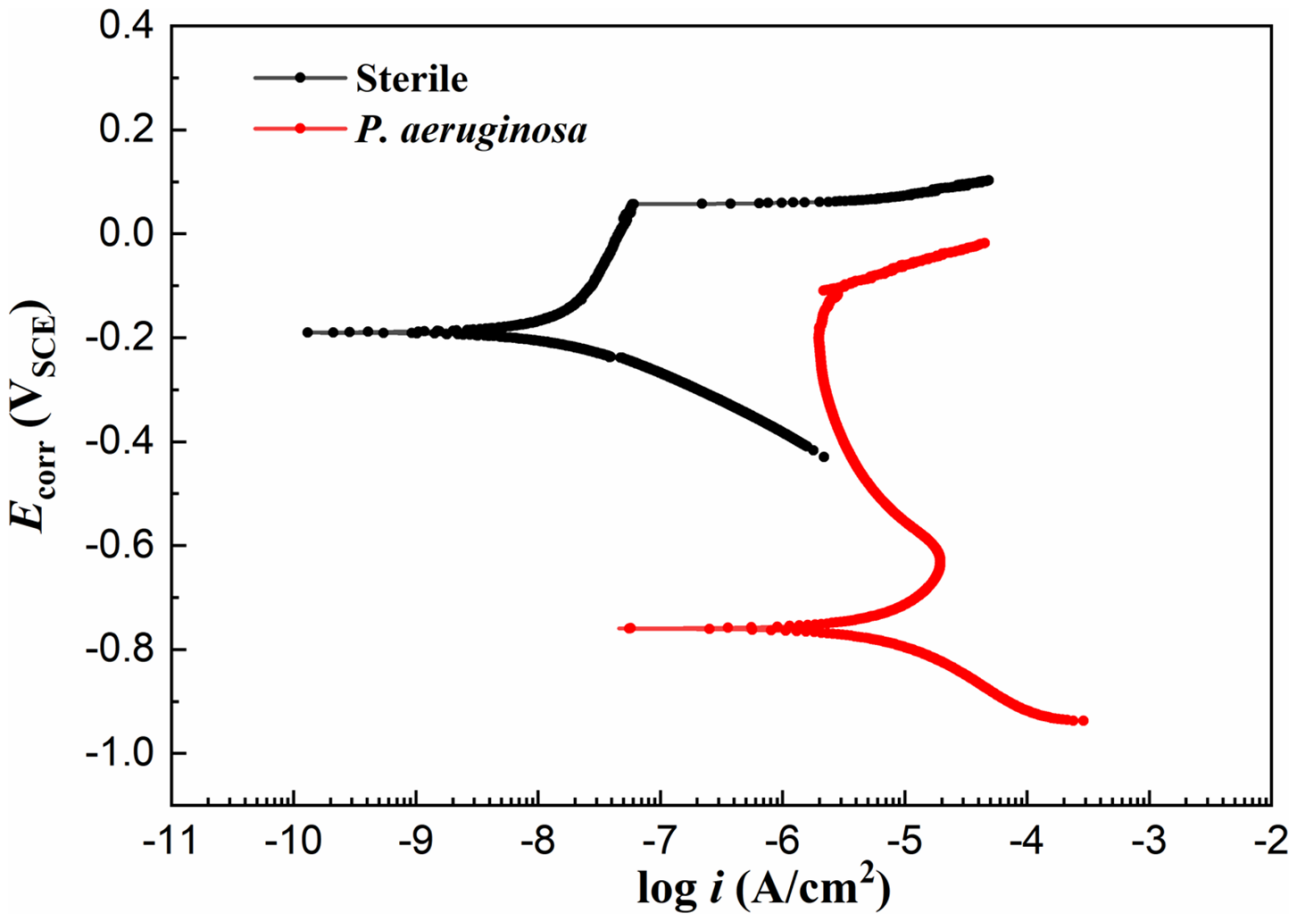
Potentiodynamic polarization curves of the HEA coupons after 14 days of immersion in sterile and *P. aeruginosa*-inoculated medium.

Figure 7 shows the potentiodynamic polarization curves of the HEA coupons after 14 days of immersion in the sterile and *P. aeruginosa*-inoculated media. The corrosion potentials (*E*_corr_) of sterile and *P. aeruginosa*-inoculated conditions were −190 mV and −759 mV, respectively. Compared with the sterile condition, the negative shift of the *E*_corr_ in the *P. aeruginosa*-inoculated medium can be attributed to the weaker passive film and local surface activation by *P. aeruginosa* metabolism. It is worth noting that the potentiodynamic polarization curve (the red curve) exhibited a significant shift to the right due to the bacterial intervention, indicating that the corrosion resistance of HEA passive film was seriously damaged.

### Surface analysis

**Figure 8:**
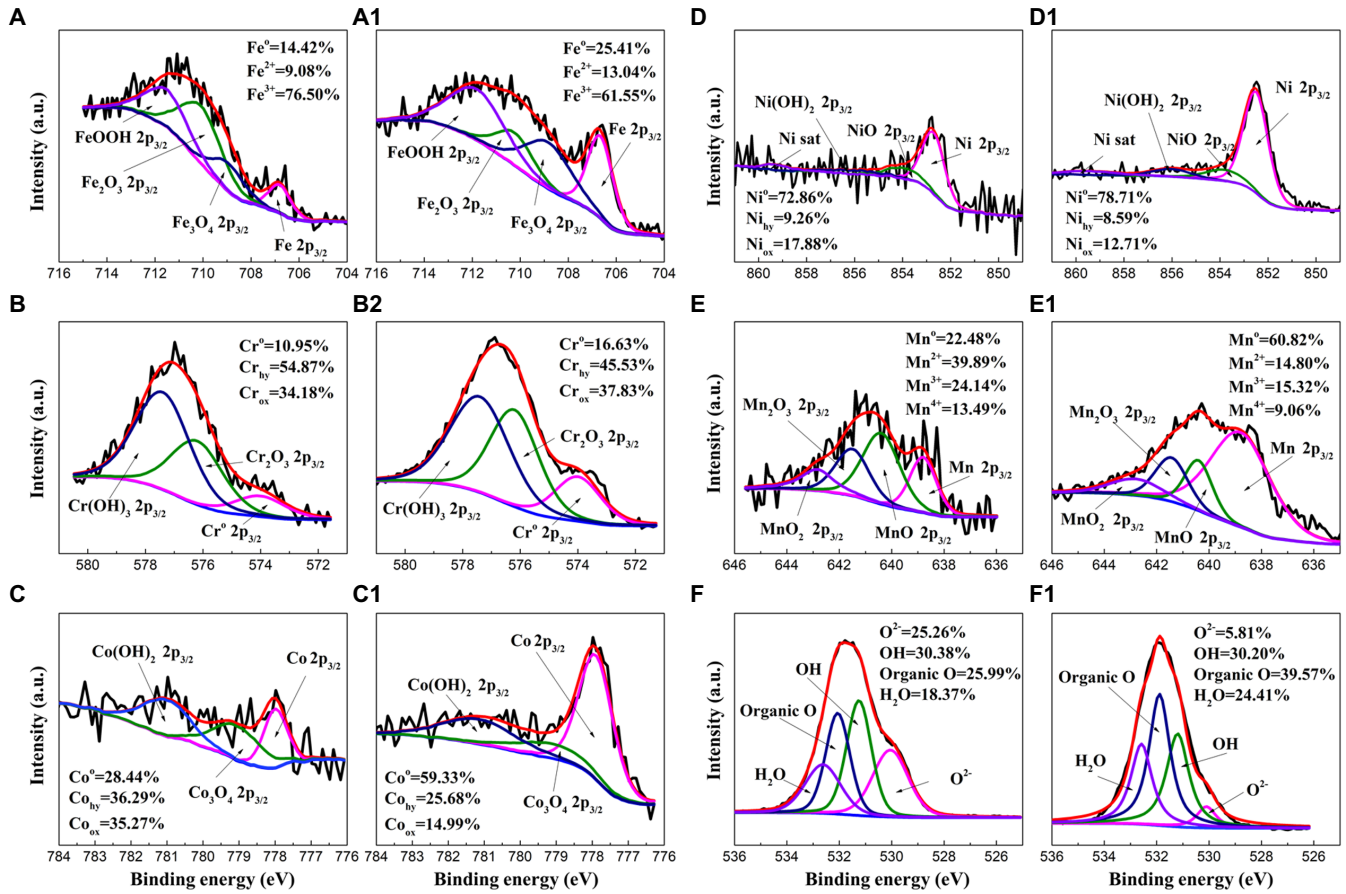
High-resolution XPS spectra of Fe_2p3/2_, Cr_2p3/2_, Co_2p3/2_, Ni_2p3/2_, Mn_2p3/2_ and O_1s_ for the HEA coupon surface after immersion in the sterile **(A–F)** and the *P. aeruginosa*-inoculated medium **(A1–F1)** for 14 days.

To further investigate the effect of *P. aeruginosa* intervention on the corrosion behavior, XPS was performed to analyze the passive film of the HEA coupons after immersion in the sterile and *P. aeruginosa*-inoculated medium for 14 days. Figure 8 shows the detailed spectra of Fe_2p3/2_, Cr_2p3/2_, Co_2p3/2_, Ni_2p3/2_, Mn_2p3/2_ and O_1s_ peaks (Xiao et al., 2020; Dai et al., 2020a,b; Zhou et al., 2021). Table 1 shows the corresponding binding energy of each compound. Fe oxides/hydroxides and Cr oxides/hydroxides are the main compounds that constitute the HEA passive film. In general, the increase of Fe^2+^/Fe^3+^ ratio is not conducive to the stability of the passive film (Dou et al., 2020; Wang et al., 2021). As shown in Figures 8A–A1, the proportions of Fe^2+^ and Fe^3+^ in the sterile medium were ~9% and ~77%, while the proportions of Fe^2+^ and Fe^3+^ in the *P. aeruginosa*-inoculated medium were ~13% and ~62%, respectively, indicating that the presence of *P. aeruginosa* promoted the significant increase of Fe^2+^ content. For other metal elements, the content of hydroxides and oxides under the sterile condition was greater than that of the *P. aeruginosa*-inoculated condition. The passive film was mainly composed of metal oxides and hydroxides, indicating that the passive film became thinner and weaker after immersion in the *P. aeruginosa*-inoculated medium for 14 days. The above results were also confirmed in the high-resolution spectra of O_1s_.

**Table 1:** Binding energies of the main components of FeCoNiCrMn HEA.

Elements	Peak	Binding energy (eV)	Elements	Peak	Binding energy (eV)
Fe 2P_3/2_	Fe metal	706.7 ± 0.1	Ni 2P_3/2_	Ni metal	852.6 ± 0.1
	Fe_2_O_3_	710.3 ± 0.1		NiO	853.9 ± 0.1
	Fe_3_O_4_	708.9 ± 0.1		Ni(OH)_2_	856.0 ± 0.1
	FeOOH	711.8 ± 0.1		Ni sat	859.3 ± 0.1
Cr 2P_3/2_	Cr metal	574.0 ± 0.1	Mn 2P_3/2_	Mn metal	638.8 ± 0.1
	Cr_2_O_3_	576.2 ± 0.1		MnO	640.4 ± 0.1
	Cr(OH)_3_	577.4 ± 0.1		Mn_2_O_3_	641.5 ± 0.1
Co 2P_3/2_	Co metal	777.9 ± 0.1		MnO_2_	642.8 ± 0.1
	Co_3_O_4_	779.0 ± 0.1	O 1s	O^2−^	530.1 ± 0.1
	Co(OH)_2_	781.0 ± 0.1		OH^−^	531.3 ± 0.1
				Organic O	532.0 ± 0.1
				H_2_O	532.6 ± 0.1

**Figure 9:**
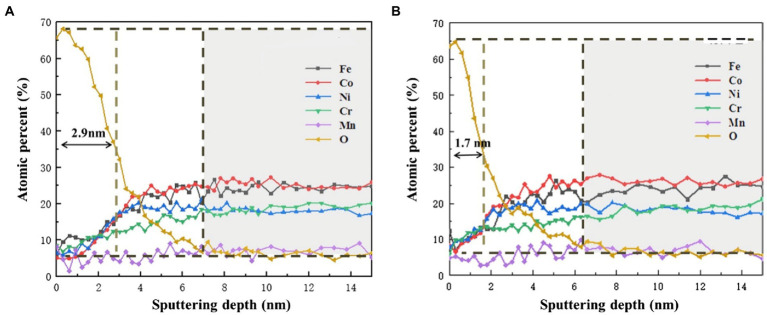
AES depth profiles of Fe, Cr, Co, Ni, Mn and O in the passive film on the HEA **(A)** in the sterile medium and **(B)** in the *P. aeruginosa*-inoculated medium.

Figure 9 shows the AES depth analysis of different elements on the surface of HEA coupons after immersion in the sterile and *P. aeruginosa*-inoculated medium for 14 days. In the sterile medium, Fe was obviously enriched on the surface of the HAE coupons. With the increasing of sputtering depth, the content of Fe gradually returned to the same level as other elements, but this phenomenon did not occur under the *P. aeruginosa*-inoculated condition. Under both sterile and *P. aeruginosa*-inoculated conditions, the O content gradually decreased with the increase of sputtering depth, indicating that the passive film on the surface of the HEA coupons was gradually broken down. In general, the thickness of the passive film was half of the sputtering depth change of O corresponding to the sputtering depth (Maurice and Marcus, 2018). Thus, the thickness of the passive film was about 2.9 nm in the sterile medium, while the thickness of the passive film in the *P. aeruginosa*-inoculated medium was about 1.7 nm which was reduced by 41%. The results were consistent with the XPS results, and also confirmed that the *P. aeruginosa* biofilm was not conducive to the formation of the HEA passive film.

**Figure 10:**
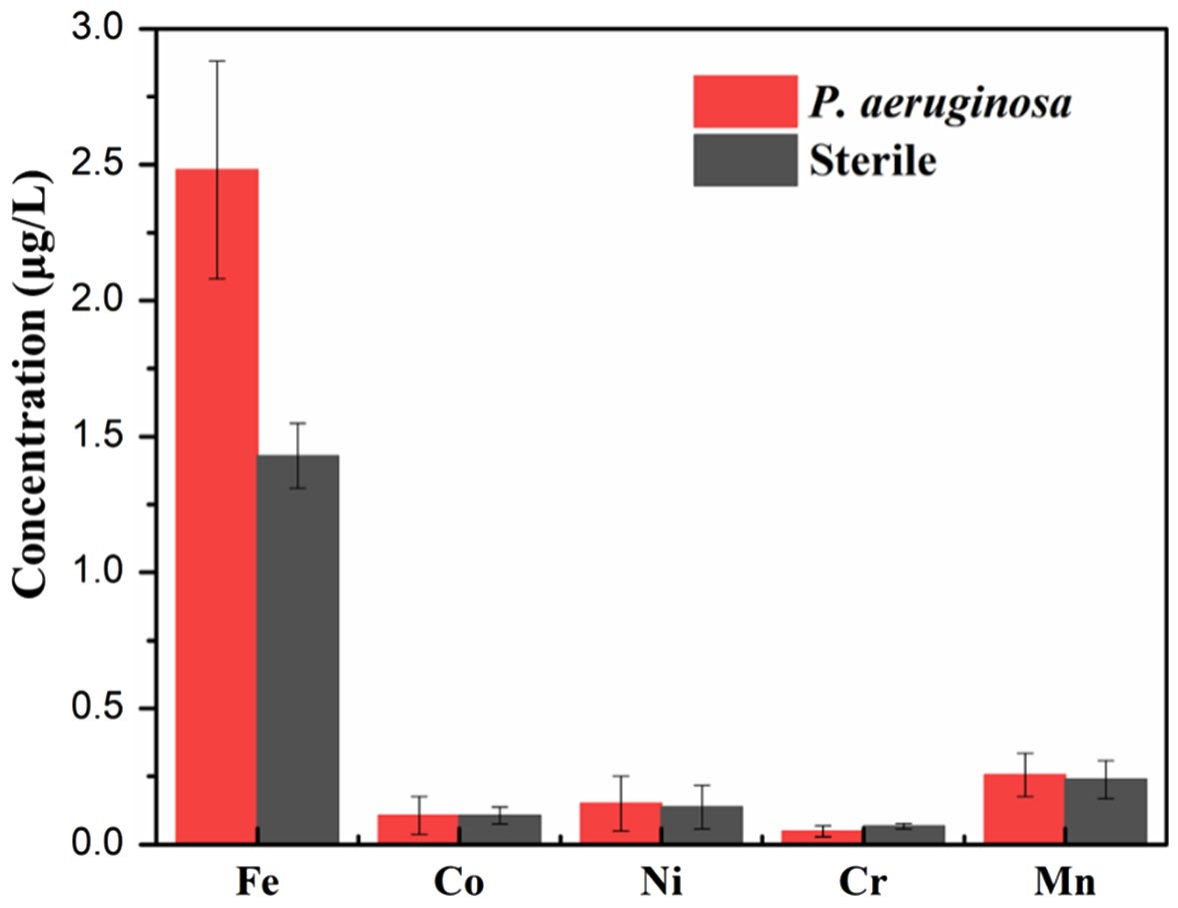
The concentrations of metallic ions dissolution from the HEA coupons in the sterile and the *P. aeruginosa*-inoculated medium after 14 days.

Figure 10 shows the variation of the ion release concentration of the HEA coupons after immersion in the sterile and the *P. aeruginosa*-inoculated medium for 14 days. Under the microbial intervention, the ions dissolution of different elements was affected. Notably, the amount of Fe dissolution was most significantly affected by microorganisms, and the release amount under the *P. aeruginosa*-inoculated condition (2.5 mg/L) was nearly twice as high as that under the sterile condition (1.4 mg/L). According to previous studies, some microorganisms can use metal Fe as an electron donor to provide energy for their growth when there are insufficient nutrients in the medium (Lou et al., 2020). Therefore, since the nutrients in the medium were consumed in the middle and later stages of the immersion experiment, *P. aeruginosa* had to make use of Fe on the HEA coupon surface for energy compensation, so as to promote the dissolution of Fe and weakened the integrality of the HEA passive film.

## Conclusion

In this work, the corrosion behavior FeCoNiCrMn high entropy alloy in the presence of *P. aeruginosa* was studied, based on surface morphology analyses such as SEM and CLSM, electrochemical analyses including OCP, LPR, EIS, potentiodynamic polarization curve as well as the analysis of corrosion products (XPS and AES), the main conclusions are as follows:

1. According to the surface analysis, a large number of biofilms were attached to the surface of the HEA coupons under the intervention of *P. aeruginosa*. The corrosion pits mostly appeared in the area covered by the biofilm, and the maximum pit depth was significantly increased.
2. The electrochemical results showed that compared with the sterile condition, the anodic reaction of HEA coupons on the *P. aeruginosa*-inoculated condition was significantly promoted, and the corrosion resistance of the passive film was significantly decreased.
3. XPS and AES results demonstrated that the *P. aeruginosa* biofilm significantly affected the thickness of the passive film and interfered with the distribution of elements in the passive film, mainly manifested as the acceleration of Fe dissolution.

## Data availability statement

The original contributions presented in the study are included in the article/supplementary material, further inquiries can be directed to the corresponding author.

## Author contributions

JY and YZ: investigation, methodology, and writing. WC: investigation and methodology. YL: conceptualization, writing, and supervision. HQ: methodology. All authors contributed to the article and approved the submitted version.

## Funding

This work was supported by the Open Fund from State Key Laboratory of Metal Material for Marine Equipment and Application (SKLMEA-K202006), the Postdoctoral Research Foundation of Shunde Graduate School of University of Science and Technology Beijing (2022BH007).

## Conflict of interest

The authors declare that the research was conducted in the absence of any commercial or financial relationships that could be construed as a potential conflict of interest.

## Publisher’s note

All claims expressed in this article are solely those of the authors and do not necessarily represent those of their affiliated organizations, or those of the publisher, the editors and the reviewers. Any product that may be evaluated in this article, or claim that may be made by its manufacturer, is not guaranteed or endorsed by the publisher.
